# The Inhibitory Action of Kohamaic Acid A Derivatives on Mammalian DNA Polymerase β

**DOI:** 10.3390/molecules14010102

**Published:** 2008-12-29

**Authors:** Yoshiyuki Mizushina, Daisuke Manita, Toshifumi Takeuchi, Fumio Sugawara, Yuko Kumamoto-Yonezawa, Yuki Matsui, Masaharu Takemura, Mitsuru Sasaki, Hiromi Yoshida, Hirosato Takikawa

**Affiliations:** 1 Laboratory of Food & Nutritional Sciences, Department of Nutritional Science, Kobe-Gakuin University, Nishi-ku, Kobe, Hyogo 651-2180, Japan; 2 Cooperative Research Center of Life Sciences, Kobe-Gakuin University, Nishi-ku, Kobe, Hyogo 651-2180, Japan; 3 Department of Applied Biological Science, Tokyo University of Science, Noda, Chiba 278-8510, Japan; 4 Faculty of Science, Tokyo University of Science, Shinjuku-ku, Tokyo 162-8601, Japan; 5 Department of Agrobioscience, Graduate School of Agricultural Science, Kobe University, Nada-ku, Kobe, Hyogo 657-8501, Japan

**Keywords:** Kohamaic acid A (KA-A), DNA polymerase (DNA-directed DNA polymerase [E.C. 2.7.7.7], pol), Enzyme inhibitor, Cytotoxicity, Computer simulation.

## Abstract

We previously isolated a novel natural product, designated kohamaic acid A (KA-A, compound **1**), as an inhibitor of the first cleavage of fertilized sea urchin eggs, and found that this compound could selectively inhibit the activities of mammalian DNA polymerases (pols). In this paper, we investigated the structure and bioactivity of KA-A and its chemically synthesized 11 derivatives (i.e., compounds **2**–**12**), including KA-A - fatty acid conjugates. The pol inhibitory activity of compound **11** [(1S*,4aS*,8aS*)-17-(1,4,4a,5,6,7,8,8a-octahydro-2,5,5,8a-tetramethyl-naphthalen-1-yl)heptadecanoic acid] was the strongest among the synthesized compounds, and the range of IC_50_ values for mammalian pols was 3.22 to 8.76 μM; therefore, the length of the fatty acid side chain group of KA-A is important for pol inhibition. KA-A derivatives could prevent human cancer cell (promyelocytic leukemia cell line, HL-60) growth with the same tendency as the inhibition of mammalian pols. Since pol β is the smallest molecule, we used it to analyze the biochemical relationship with KA-A derivatives. From computer modeling analysis (i.e., docking simulation analysis), these compounds bound selectively to four amino acid residues (Leu11, Lys35, His51 and Thr79) of the N-terminal 8-kDa domain of pol β, and the binding energy between compound **11** and pol β was largest in the synthesized compounds. The relationship between the three-dimensional molecular structures of KA-A-related compounds and these inhibitory activities is discussed.

## Introduction

A novel sesterterpenic acid, kohamaic acid A (KA-A), was isolated from a marine sponge *Ircinia* sp. [1]. KA-A was first screened as an inhibitor of the first cleavage of fertilized sea urchin eggs [1], but we also found and reported that it inhibited the activities of DNA polymerases (pols) from the deuterostome branch in the phylogenetic tree, but not from plants, prokaryotes, or even protostomes such as insects and mollusks [2]. 

Pol is associated with genomic DNA replication, repair and recombination in eukaryotic cells. Eukaryotic cells reportedly contain three replicative types; pols α, δ, and ε, mitochondrial pol γ, and at least twelve repair types; pols β, δ, ε, ζ, η, θ, ι, κ, λ, μ, and σ and REV1 [3]. The roles of the pols have not yet been fully established. Against this background, it is of interest that KA-A could only inhibit the activities of the deuterostome pols tested, including those of the sea urchin.

**Figure 1:**
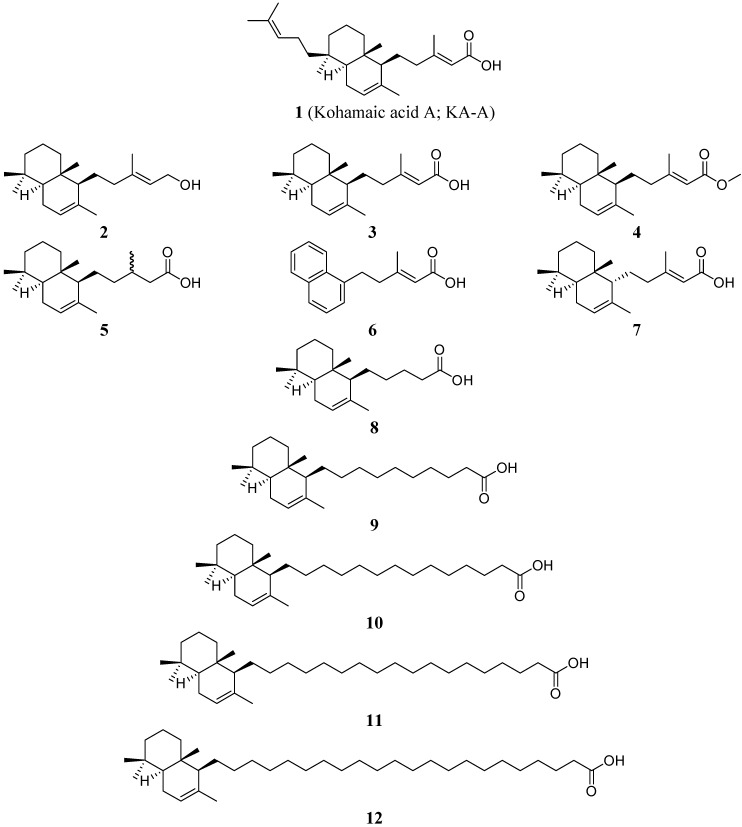
Structures of kohamaic acid A and its derivatives.

Pols α and β have been isolated and characterized from sea urchins [4, 5]. The possible role of cell multiplication in sea urchin gastrulation has been somewhat neglected and its importance is in general considered secondary [6]; however, it is well known that DNA synthesis continues during gastrulation [7, 8] and that the cell number increases about three times between the hatched blastula and the prism stage [7]. It has also been reported that primary mesenchyme cells undergo mitotic divisions after they have been shed into the blastocoel [9]. This indirect evidence suggests that KA-A is useful for investigating the relationship between sea urchin pols and the cleavage of fertilized sea urchin eggs, and that KA-A may induce inhibition of the first cleavage of fertilized sea urchin eggs by inhibiting DNA replication [2]; subsequently, we succeeded in chemically synthesizing KA-A (compound **1**) and its eleven derivatives (compounds **2**–**12**) (Figure 1) [10]. 

In this report, we investigated the inhibitory activities of mammalian pols and human cancer cell growth for the development of anticancer chemotherapy drugs, because the inhibition of pols will lead to cell death, especially under proliferation conditions such as in cancer cells; therefore, inhibitors of eukaryotic pols should be considered as potential agents for cancer chemotherapy. We also discuss the molecular inhibition mechanism of pol β activity by KA-A derivatives. 

Compound **1**, kohamaic acid A (KA-A); compound **2**, (5S*,9S*,10S*,13*E*)-labda-7,13-dien-15-ol; compound **3**, (5S*,9S*,10S*,13*E*)-labda-7,13-dien-15-oic acid; compound **4**, methyl (5S*,9S*,10S*,13*E*)-labda-7,13-dien-15-oate; compound **5**, (5S*,9S*,10S*,13R*S*,13*E*)-labda-7-en-15-oic acid; compound **6**, (*E*)-3-methyl-5-(naphthalen-1-yl)-2-pentenoic acid; compound **7**, (5S*,9S*,10R*,13*E*)-labda-7,13-dien-15-oic acid; compound **8**, (1S*,4aS*,8aS*)-5-(1,4,4a,5,6,7,8,8a-octahydro-2,5,5,8a-tetramethylnaphthalen-1-yl)pentanoic acid; compound **9**, (1S*,4aS*,8aS*)-9-(1,4,4a,5,6,7,8,8a-octahydro-2,5,5,8a-tetramethylnaphthalen-1-yl)nonanoic acid; compound **10**, (1S*,4aS*,8aS*)-13-(1,4,4a,5,6,7,8,8a-octahydro-2,5,5,8a-tetramethylnaphthalen-1-yl)tridecanoic acid; compound **11**, (1S*,4aS*,8aS*)-17-(1,4,4a,5,6,7,8,8a-octahydro-2,5,5,8a-tetramethylnaphthalen-1-yl)heptadecanoic acid; and compound **12**, (1S*,4aS*,8aS*)-21-(1,4,4a,5,6,7,8,8a-octahydro-2,5,5,8a-tetramethylnaphthalen-1-yl)henicosanoic acid.

## Results and Discussion

### Effects of KA-A derivatives on the activities of mammalian DNA polymerases α and β

As briefly described in the Introduction, we found and reported that KA-A (compound **1**) is an inhibitor of pols only from the deuterostome branch in the phylogenetic tree, including mammals [2]. The purpose of this study was to investigate the inhibitory mechanism more precisely using eleven chemically synthesized derivatives of KA-A (compounds **2**–**12**), which were prepared as described previously [10]. The chemical structures of KA-A and its analogs are shown in Figure 1. 

**Figure 2:**
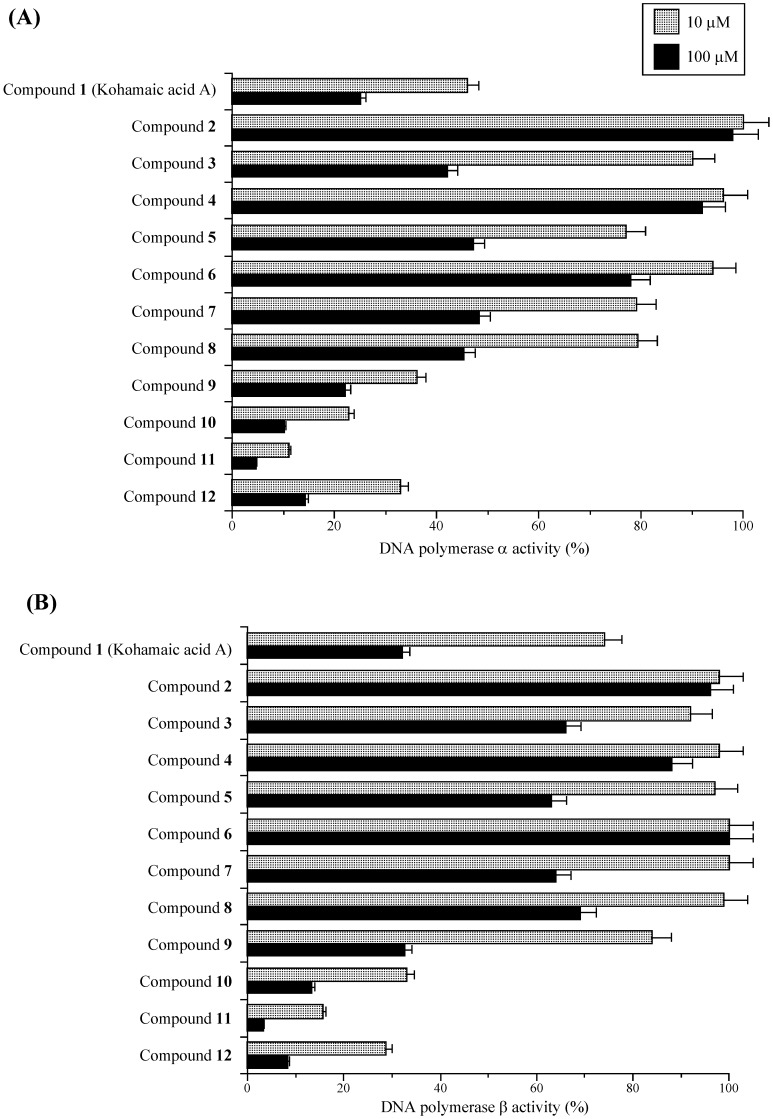
Effects of kohamaic acid A derivatives (compounds **1**–**12**) on the activities of mammalian DNA polymerases α and β.

First, the relative activities of calf pol α and rat pol β with two set concentrations (10 and 100 μM) of the test compounds are shown in Figure 2. Pol α and pol β were used as representative replicative pol and repair/recombination-related pol, respectively [11, 12]. As reported previously, KA-A dose-dependently inhibited the activities of pols α and β, and the IC_50_ values were 7.6 and 8.4 μM, respectively [2]. In the synthesized compounds (i.e., compounds **2**–**12**), compounds **2** to **8** were weaker inhibitors of pols α and β than KA-A, and the inhibitory effect of compound **9** was as strong as that of KA-A, whereas, compounds **10**–**12** were stronger inhibitors than KA-A, and compound **11** had the strongest inhibitory effect on pols α and β of all the compounds tested. The inhibition of pol α activity by the compounds showed the same tendency as that of pol β activity. KA-A derivatives, such as compounds **8** to **12**, have a conjugated fatty acid in the KA-A molecule, and we previously reported that longer chain fatty acids inhibit the activity of eukaryotic pols; therefore, the fatty acid region of compounds **10**–**12** must be important for inhibition. 

Each compound [10 M, (gray bar) and 100 M (black bar)] was incubated with calf pol α (A) and rat pol β (B). Pol activity was assayed as described previously [38, 39]. Enzyme activity in the absence of compounds was taken as 100 %. All data are shown as the means ± SEM of three independent experiments.

### Effects of KA-A derivatives on cultured human cancer cells

Interest also focused on developing agents for cancer chemotherapy using these inhibitors. Replicative pols, such as pol α, are regarded as potential targets of anticancer drugs, because they play central roles in DNA replication, which is indispensable for the proliferation of cancer cells. KA-A derivatives could therefore be useful in chemotherapy, and we investigated the cytotoxic effect of KA-A and its related compounds (i.e., compounds **1** to **12**) against a human promyelocytic leukemia cell line, HL-60. 

**Figure 3:**
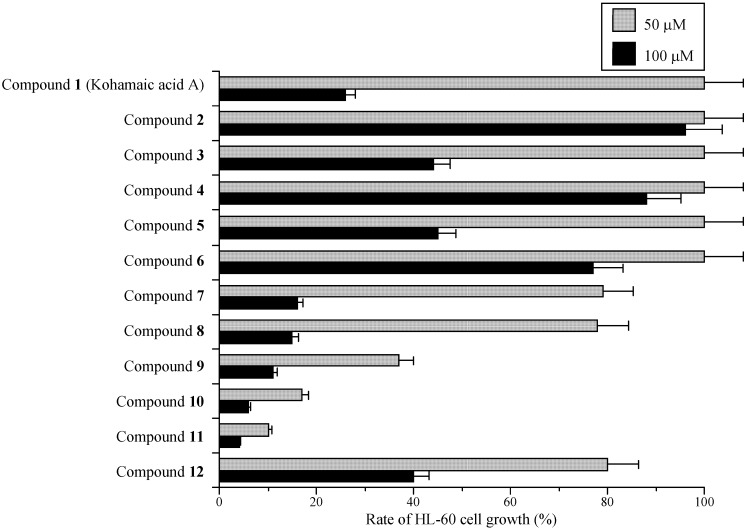
Effects of kohamaic acid A derivatives (compounds **1**–**12**) on proliferation of HL-60 cancer cells.

As shown in Figure 3, 50 μM of compound **11** had the strongest growth inhibitory effect on HL-60 cells of the compounds tested, compounds **12** and **10** were the second and third strongest, respectively. Cell growth suppression had the same tendency as the inhibition of mammalian pols α and β among the compounds, suggesting that KA-A derivatives are able to penetrate cancer cells and reach the nucleus, inhibiting pol activity (Figure 2 and Figure 3). We therefore concentrated our efforts on (1S*,4aS*,8aS*)-17-(1,4,4a,5,6,7,8,8a-octahydro-2,5,5,8a-tetramethylnaphthalen-1-yl)heptadecanoic acid (**11**) in subsequent experiments. 

Each compound (50 and 100 μM each) was incubated with human cancer cells (i.e., promyelocytic leukemia cell line, HL-60). Cell proliferative growth was measured by MTT (3-(4,5-dimethylthiazol-2-yl)-2,5-diphenyl tetrazolium bromide) assay [41]. The growth rate of cancer cells in the absence of compounds was taken as 100 %. All data are shown as the means ± SEM of five independent experiments.

### Inhibitory effect of compound **11** on the activities of DNA polymerases and other DNA metabolic enzymes

As shown in Table 1, compound **11** inhibited the activities of all the mammalian pols tested, and the range of the IC_50_ values was 3.22–8.76 μM. The inhibitory effect on human pol ε was the strongest of the mammalian pols tested. Given that the pol A family includes pol γ, the pol B family includes pols α, δ and ε, the pol X family includes pols β and λ, and the pol Y family includes pols η, ι and κ [13,14,15], compound **11** could inhibit the activities of all families of mammalian pols and fish pols such as cherry salmon pol δ. On the other hand, the activity of plant pols such as cauliflower pol α, prokaryotic pols such as the Klenow fragment of *E. coli* pol I, *Taq* pol and T4 pol, and DNA metabolic enzymes such as calf primase of pol α, T7 RNA polymerase, T4 polynucleotide kinase and bovine deoxyribonuclease I (DNase I), were not influenced by compound **11**. When activated DNA was used as the DNA template-primer instead of poly(dA)/oligo(dT)_12-18_, the inhibition modes of these compounds did not change (data not shown). These results suggest that compound **11** should be classified as an inhibitor of mammalian pols. 

**Table 1:** IC_50_ values of compound **11** on the activities of various DNA polymerases and other DNA metabolic enzymes.

Enzyme	IC_50_ values (μM)
Mammalian DNA polymerases	
Calf DNA polymerase α	4.21 ± 0.21
Rat DNA polymerase β	5.50 ± 0.28
Human DNA polymerase γ	8.76 ± 0.44
Human DNA polymerase δ	4.89 ± 0.24
Human DNA polymerase ε	3.22 ± 0.16
Human DNA polymerase η	7.45 ± 0.37
Human DNA polymerase ι	7.84 ± 0.39
Human DNA polymerase κ	7.20 ± 0.36
Human DNA polymerase λ	4.67 ± 0.23
Fish DNA polymerase	
Cherry salmon DNA polymerase δ	4.30 ± 0.22
Plant DNA polymerase	
Cauliflower DNA polymerase I (α-like)	>200
Prokayotic DNA polymerases	
*E. coli* DNA polymerase I (Klenow fragment)	>200
*Taq* DNA polymerase	>200
T4 DNA polymerase	>200
Other DNA metabolic enzymes	
Calf Primase of DNA polymerase α	>200
T4 Polynucleotide kinase	>200
Bovine Deoxyribonuclease I	>200

Compound **11** was incubated with each pol (0.05 units) and other DNA metabolic enzymes. One unit of pol activity was defined as the amount of enzyme that catalyzed the incorporation of 1 nmol of dNTP (i.e., dTTP) into the synthetic DNA template-primers (i.e., poly(dA)/oligo(dT)_12-18_, A/T = 2/1) in 60 min at 37 °C under normal reaction conditions for each enzyme. Enzyme activity in the absence of the compounds was taken as 100%. Data are shown as the means ± SEM of four independent experiments.

### Effect of interaction of nucleic acid, protein and compound **11**

To determine whether the inhibition resulted in binding to DNA or enzymes, the interaction of compound **11** with double-stranded DNA (dsDNA) was investigated based on the thermal transition of dsDNA with or without compound **11**. The Tm of dsDNA with an excess amount of compound **11** (200 μM) was measured using a spectrophotometer equipped with a thermoelectric cell holder. In the concentration range used, no thermal transition of Tm was observed, whereas ethidium bromide used as a positive control, a typical intercalating compound, produced clear thermal transition (data not shown). These results indicate that compound **11** does not intercalate to DNA as a template-primer, and the compound may directly bind to the enzyme and inhibit its activity. 

**Table 2:** Effects of poly (rC), bovine serum albumin (BSA) or Nonidet P-40 (NP-40) on the inhibition of rat DNA polymerase β activity by compound **11**.

Compounds added to the reaction mixture	Relative activity of pol β (%)
Without compound **11**	
None (control)	100 ± 5.0
+ 50 μM poly (rC)	100 ± 4.6
+ 200 μg/ml BSA	100 ± 8.9
+ 0.05 % NP-40	100 ± 5.9
+ 0.1 % NP-40	100 ± 8.5
10 μM compound **11**	
10 μM compound **11**	15.5 ± 0.78
10 μM compound **11** + 50 μM poly (rC)	15.2 ± 0.74
10 μM compound **11** + 200 μg/ml BSA	15.9 ± 1.1
10 μM compound **11** + 0.05 % NP-40	96.1 ± 7.6
10 μM compound **11** + 0.1 % NP-40	100 ± 8.8
100 μM compound **11**	
100 μM compound **11**	3.2 ± 0.16
100 μM compound **11** + 50 μM poly (rC)	3.1 ± 0.15
100 μM compound **11** + 200 μg/ml BSA	3.3 ± 0.19
100 μM compound **11** + 0.05 % NP-40	62.5 ± 4.1
100 μM compound **11** + 0.1 % NP-40	95.0 ± 7.6

50 μM poly (rC), 200 μg/ml BSA, 0.05% NP-40 or 0.1% NP-40 were added to the reaction mixture. In the absence of compound **11**, DNA polymerase activity was taken as 100%. Data are shown as the means ± SEM of four independent experiments.

To determine the effects of a non-ionic detergent on the binding of compound **11** to rat pol β, Nonidet P-40 (NP-40) was added to the reaction mixture at a concentration of 0.05 or 0.1% (Table 2). In the absence of compound **11**, the activity of pol β was not affected by the addition of NP-40, and we designated the activity in these cases as 100%. The inhibitory effect of compound **11** at 10 and 100 μM was completely reversed by the addition of 0.1% NP-40 to the reaction mixture. These results suggest that compound **11** can bind to and interact with the hydrophobic region of the enzyme protein. We also tested whether an excess amount of a substrate DNA analog, poly(rC) (50 μM), or a protein, BSA (200 μg/ml), could prevent the inhibitory effects of compound **11**. If the compound binds to the enzymes by non-specific adhesion, the addition of the nucleic acid and/or protein will be expected to reduce inhibitory activity. Neither poly(rC) nor BSA influenced the inhibitory effects on compound **11**, suggesting that the compound can interact selectively or bind to a specific site on pol β and not to the substrate (i.e., nucleic acid). These results for compound **11** were obtained using mammalian pols other than pol β (data not shown). 

Pol β has the smallest molecular weight (i.e., 39-kDa) of all eukaryotic pols, and the three-dimensional structure of pol β was determined [16, 17]; therefore, we focused on analyzing the biochemical and molecular mechanism of pol β inhibition by compound **11** in the latter part of this study. 

### Mode of inhibition of DNA polymerase β by compound **11**

Next, to elucidate the mechanism by which compound **11** inhibited pol β, the extent of inhibition as a function of substrate concentration was studied. In kinetic analysis of pol β, poly(dA)/oligo(dT)_12-18_ (molecular concentration of primer 3’-ends) and 2'-deoxythymidine 5'-triphosphate (dTTP) were used as the DNA template-primer and 2’-deoxyribonucleotide 5’-triphosphate (dNTP) substrate, respectively. As shown in Table 3, double reciprocal plots (Lineweaver Burk plots) of the results showed that the compound **11**-induced inhibition of rat pol β activity was competitive with respect to the DNA template-primer, because the apparent maximum velocity (Vmax) was unchanged at 111 pmol/h, whereas 22.0 pmol/h of the Michaelis constant (Km) increased in the presence of 9 μM of compound **11**. The inhibition mode was also competitive with respect to the dNTP substrate, and the Vmax for the dNTP substrate was unchanged at 62.5 pmol/h, and the Km for the dNTP substrate increased from 3.05 to 10.8 μM in the presence of 9 μM of compound **11**. The inhibition constant (Ki) values, obtained from Dixon plots, were found to be 1.96 μM and 2.36 μM for the DNA template-primer and dNTP substrate, respectively. 

**Table 3:** Kinetic analysis of the inhibitory effects of compound **11** on the activities of rat DNA polymerase β as a function of the DNA template-primer dose and the nucleotide substrate concentration.

Enzyme	DNA Substrate	Compound 11 (μM)	Km ^a)^ (μM)	Vmax ^a)^ (pmol / h)	Ki ^b)^ (μM)	Inhibitory mode ^a)^
**Pol β**	Template	0	6.74	111	1.96	Competitive
	-primer ^c)^	3	8.77			
		6	12.7			
		9	22.0			
	Nucleotide ^d)^	0	3.05	62.5	2.36	Competitive
	substrate	3	4.03			
		6	5.81			
		9	10.8			

^a)^ These data were obtained from Lineweaver Burk plot. ^b)^ These data were obtained from Dixon plot. ^c)^ poly(dA)/oligo(dT)_12-18_. ^d)^ dTTP.

The inhibition of pol β by compound **11** had the same kinetic mode as that of other pol X family members, such as pol λ, i.e., competitive with respect to both the DNA template-primer and the dNTP substrate, suggesting that the compound can bind directly to both the DNA template-primer-binding site and the dNTP substrate-binding site, and may directly inhibit the DNA polymerization process. As the Ki values for nucleic acid were similar to those for the dNTP substrate, the affinity of compound **11** and the enzyme-nucleic acid may be the same as that of compound **11** and the enzyme-nucleotide substrate. 

### Three-dimensional modeling of the interaction of KA-A derivatives with DNA polymerase β

**Figure 4:**
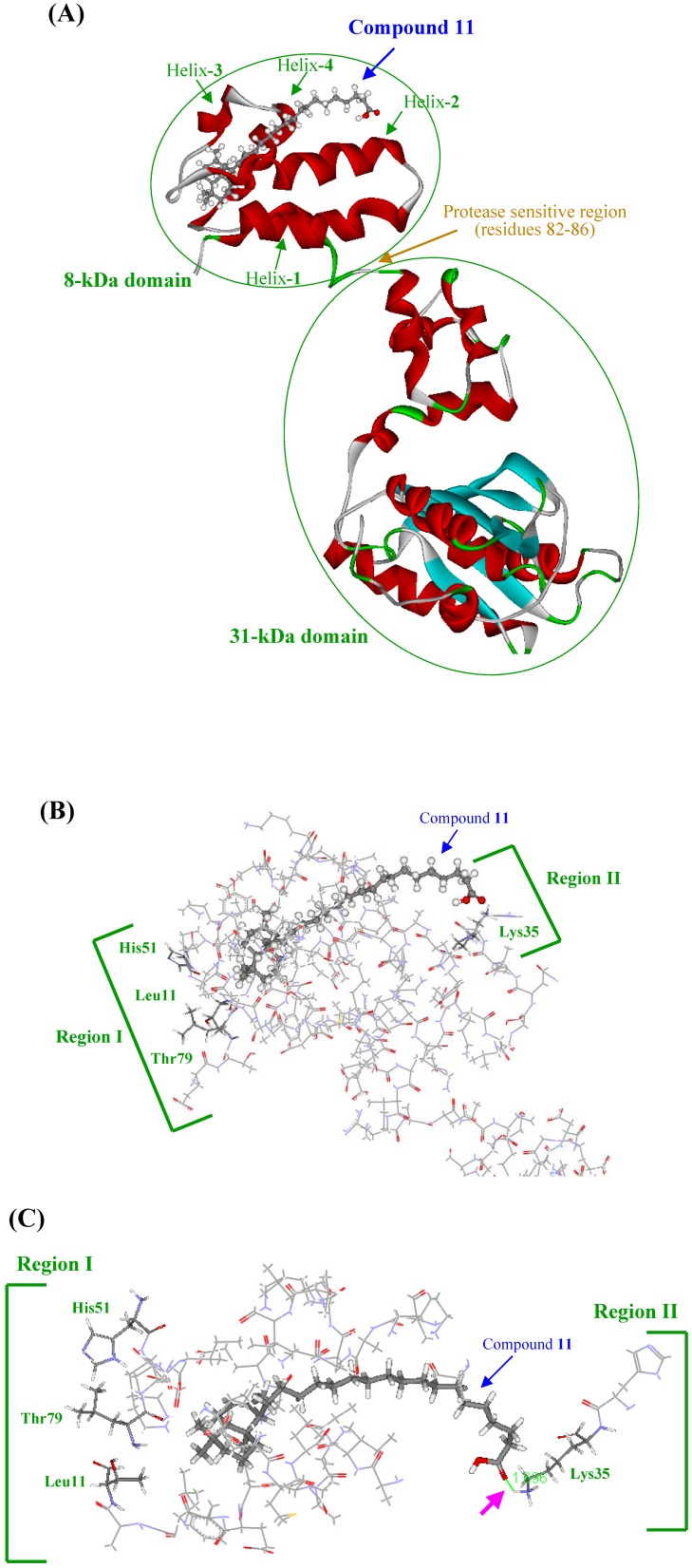
Docking simulation of compound **11** with rat DNA polymerase β.

Further investigations of the three-dimensional structure of the binding site on pol β and the modes of binding of compound **11** are necessary. We previously reported the characteristics of binding between pol β and linear-long chain fatty acids (for example: C24-nervonic acid), which are components of KA-A derivative compounds **7**–**11**, involved in the inhibition of pol β activity [18]. Pol β is the smallest known pol in animal cells with a molecular mass of 39-kDa, and its structure is highly conserved among mammals [19]. This protein has a modular two-domain structure, with apparent flexibility within a protease-sensitive region between residues 82–86, which separates the two domains, an N-terminal domain fragment (8-kDa), which retains binding affinity for single-stranded DNA (ssDNA), and a C-terminal domain fragment (31-kDa) with reduced pol activity (Figure 4A) [20, 21]. The linear-long chain fatty acids bound to pol β at the N-terminal 8-kDa domain, where they competed with the DNA template-primer [18, 22]. One molecule of each of the agents in the fatty acid region competed with one molecule of DNA template-primer, and subsequently interfered with the binding of a DNA template-primer to one 8-kDa domain, indicating that the 8-kDa domain fragment bound to the fatty acids as a 1:1 complex. Biochemical and surface plasmon resonance (BIAcore) demonstrated that KA-A derivatives, including compound **11**, bound selectively to the *N*-terminal 8-kDa domain of pol β at a molecular ratio of 1 : 1, this compound inhibited ssDNA binding activity, and the binding of these compounds indirectly inhibited catalytic activity on the 31-kDa domain (data not shown). 

The NMR structure of the *N*-terminal 8-kDa domain of pol β has been determined by Wilson, Mullen and their co-workers [22]. According to their results, the 8-kDa domain (residues 1–87) is formed by four α-helices, packed as two antiparallel pairs. The pairs of α-helices cross one another at 50°, giving them a V-like shape. The 8-kDa domain contains a motif termed the "Helix-hairpin-Helix" (HhH). The protein residues involved in template DNA-binding have been identified by NMR using chemical shift changes [23]. The Helix-3-hairpin-Helix-4 motif and residues in an adjacent W-type loop connecting helix-1 and helix-2 form the ssDNA interaction surface [24]. Furthermore, it was also found that several mutants of the 8-kDa domain (F25W, K35A, K60A and K68A) showed impaired template DNA-binding activity [25]. The structure of the 8-kDa domain fragment with linear-long chain fatty acids has been determined by multi-dimensional NMR in more detail [18]. The interactions with the fatty acids were mapped to one face of the fragment by characterizing backbone ^1^H and ^15^N chemical shift changes. In the 8-kDa domain fragment with linear-long chain fatty acids, the structure that forms the interface included helix-1, helix-2, helix-4, a turn (residues from 48 to 51) and residues adjacent to an Ω-type loop connecting helix-1 and helix-2. Since the alkyl chain group of the fatty acids appears to bind to Leu11, His51 and Thr79 of the interface on amino acid sheets of the 8-kDa domain fragment and the carboxyl group interacts with the Lys35 site, the distance between the alkyl chain end and the carboxyl end might be important for tight binding. Only the shifted cross-peaks of Leu11 and Thr79 were significantly changed by the length of the carbon chain. Longer fatty acids could bind to the fragment more tightly. 

(A) Interaction between compound **11** and rat pol β. The Cα-backbone, α-helix and β-sheet in pol β are shown in green, red and light-blue, respectively. (B and C) Interaction between compound **11** and the 8-kDa domain of pol β. The amino acid residues of the 8-kDa domain are shown as stick models, and carbons, nitrogens, oxygens and sulfurs are in gray, light-blue, red and yellow, respectively. The calculations used an ESFF force-field in the Discovery program. Amino acid residues Leu11, Lys35, His51 and Thr79, which bind to linear-long chain fatty acids, are indicated. The molecular docking of compound **11** and the 8-kDa domain of rat pol β was modeled using the affinity program of Insight II modeling software (Accelrys Inc., San Diego, CA, USA, 1999). The carbons, hydrogens and oxygens of compound **11** are in gray, white, and red, respectively. The Protein Data Bank (PDB) codes of pol β and the 8-kDa domain are 1BPD and 1BNO, respectively. This figure is presented using PyMol (DeLano Scientific, San Carlos, CA, USA, 2002).

Based on these results, the binding of KA-A derivatives containing fatty acid (i.e., compounds **7**–**12**) to the three-dimensional structure of rat pol β was simulated by utilizing the above information, and the interaction interface of these compounds on the amino acid sheets of the 8-kDa domain fragment was mostly the same as in linear-long chain fatty acids (for example: C24-nervonic acid). In the energy-minimized docking simulation, compound **11** was the strongest binding energy among the compounds tested, and the binding force (-106.53 kcal/mol) consisted of Coulomb force (-94.68 kcal/mol) and van der Waals force (-11.85 kcal/mol) (Table 4). In the order of binding energy, these KA-A-related compounds ranked as follows: compound **11** > compound **12** > compound **10** > compound **9** > compound **8** > compound **7**. This energy of KA-A derivatives containing fatty acids showed the same tendency as the inhibitory activity of pol β (Figure 2B). 

**Table 4:** Binding energy of kohamaic acid A derivatives (compounds **7**–**12**) and the 8-kDa domain of DNA polymerase β.

Compound	Energy (kcal/mol)		
	Coulomb	van der Waals	Total
**7**	-12.81	-6.89	-19.70
**8**	-4.48	-7.79	-12.27
**9**	-56.61	-9.44	-66.05
**10**	-65.42	-10.20	-75.62
**11**	-94.68	-11.85	-106.53
**12**	-66.14	-17.23	-83.37

The 8-kDa domain of rat DNA polymerase β (residues 2–88, PDB code; 1BPD) with each KA-A derivatives (compounds **7**–**12**) is indicated. Binding energy was calculated by the flexible docking procedure in the affinity program within the Insight II modeling software (Accelrys Inc., San Diego, CA, USA).

The molecular lengths of these compounds are shown in Table 5. Since compound **11** was the strongest inhibitor of pol β in compounds **7**–**12**, the molecular length of compound **11** (i.e., 17.47 Å) might best fit the pocket of the 8-kDa domain. 

**Table 5:** The molecular length and wide of three-dimensional structure of kohamaic acid A derivatives (compounds **1**–**12**).

Compound	Length (Å)	Wide (Å)
**1** (kohamaic acid A)	16.76	6.45
**2**	11.96	6.45
**3**	12.08	6.45
**4**	13.92	6.45
**5**	11.42	6.45
**6**	11.28	5.10
**7**	11.51	6.45
**8**	11.57	6.45
**9**	17.47	6.45
**10**	22.31	6.45
**11**	26.69	6.45
**12**	31.61	6.45

The energy-minimized three-dimensional molecular structures of KA-A and its derivatives (compounds **1**–**12**) were prepared using Insight II (Accelrys, San Diego, CA, USA), and the maximum length and width of the compounds were measured.

Therefore, docking simulation of compound **11** and the 8-kDa domain of pol β is shown in Figure 4, and the compound **11** binding interface of the 8-kDa domain having the same pocket (i.e., the crevice between helix-1 and helix-2) as linear-long chain fatty acids (Figure 4A). This result suggested that Lys35, which is a hydrophilic amino acid in "region II", bound to the carboxyl group of compound **11**, and Leu11 and His51, which are hydrophobic amino acids in "region I", bound to the bicyclic core part of compound **11** (Figure 4B). Prasad *et al*. reported that DNA template [i.e., p(dT)_8_] binding activity was impaired in site-directed mutants of Phe25, Lys35, Lys60 or Lys68 [25]. Region II containing Lys35, shown in Figure 4B, appears to have an important role in the effect of compound **11**. This compound probably competes with the DNA template-primer at residue Lys35 and binds to the site, which subsequently inhibits ssDNA-binding activity in the 8-kDa domain. In region I shown in Figure 4B, Leu11, His51 and Thr79 are different from other DNA-binding sites (i.e., Phe25, Lys60 and Lys68), suggesting that the hydrophobic moieties of the alkyl chain and bicyclic core part in compound **11** do not disturb the binding of the DNA template-primer, and these hydrophobic amino acids in region I must be important for binding to the compound by hydrophobic force. In this docking simulation, the carboxyl group of compound **11** and the residue of Lys35 made a hydrogen bond (pink arrow in Figure 4C). The carboxyl group in compound **11** is thought to be important for the inhibition of pol β, because the compound modified from the carboxyl group to a methyl ester could not inhibit activity (data not shown). The distance between the Lys35 hydrophilic region and Leu11 and His51 hydrophobic regions fits the length of the U-shaped compound **11**, and intercalated smoothly into the pocket between helix-1, 2 and helix-3, 4; therefore, both the three-dimensional molecular length and the carboxyl end of KA-A derivatives are important for both fitting to bind the pocket and the inhibitory activity of DNA polymerization and ssDNA binding on the 8-kDa domain of pol β. The three-dimensional structural binding analysis between the 8-kDa domain fragment of pol β and compound **11** will be measured using the multi-dimensional (^1^H-^15^N HMQC) NMR for further study. 

Although the polymerase active site of pol β is homologous to other pols, it structure is unique to family X pols [3]. These results suggested that other pols from alternate families (e.g., families A, B and Y) might have similar binding sites even though they do not have an equivalent domain. 

Drug design will be possible by investigating the tightness of the binding between the KA-A derivatives and pol β. Based on information available from NMR analysis, computer simulation of the conformational changes in pol β with or without newly designed KA-A related compounds will be useful for this purpose. 

We have been screening for new pol inhibitors to use for analyzing the structure and function of mammalian pols to understand their precise roles *in vivo*, and to develop drug design strategies for the development of cancer chemotherapy agents. These inhibitors are not only molecular tools for analyzing pols, but are also potentially useful for cancer chemotherapy. Subsequently, we found that KA-A (compound **1**) was a potentially useful agent [2], and synthesized KA-A derivatives (compounds **2** – **12**) [10]. The inhibitory effect of compound **11** on both mammalian pol activity and human cancer cell growth was strongest of all compounds tested (Figure 2 and Figure 3), and this compound inhibited enzyme activity at an IC_50_ of 4.21 μM for pol α and 5.50 μM for pol β (Table 1). This compound showed markedly stronger inhibitory effects on pol α than aphidicolin (IC_50_ = 40 μM) (data not shown). We should also emphasize that compound **11** is a 4-fold stronger pol β inhibitor than dideoxyTTP, a potent inhibitor of pol β [26]. Due to their strong inhibitory effects, these KA-A derivatives could be useful as pol inhibitors. 

In this report, we explained the structure-function relationship in the inhibition of pols by synthetic derivatives of KA-A, and elucidated the molecular mechanism of the inhibitory action of these compounds on pol activity and mammalian cell proliferation. In compound **11**, not only the fatty acid region but the bicyclic core part might have an important role in the inhibition of pol activity. The molecular mechanism of the inhibition seemed to be dependent on the fatty acid chain length. 

In this study, we discussed the pharmaceutical potential of KA-A derivatives such as compound **11** as inhibitors of mammalian pol and human cancer cell growth. This will be a useful approach to finding and developing future therapeutic drugs. 

## Experimental

### General

Except for natural KA-A (compound **1**), all derivatives (compounds **2**–**12**) were synthesized in a non-enantioselective manner [10]. The chemical structures of the compounds are shown in Figure 1. Nucleotides and chemically synthesized DNA template-primers, such as poly(dA) and oligo(dT)_12-18_, and radioisotope reagents such as [^3^H]-dTTP (43 Ci/mmol) were purchased from GE Healthcare Bio-Science Corp. (Buckinghamshire, UK). All other reagents were of analytical grade and were purchased from Nacalai Tesque, Ltd. (Kyoto, Japan). 

### Enzymes

Pol α was purified from calf thymus by immuno-affinity column chromatography as described by Tamai *et al*. [27]. Recombinant rat pol β was purified from *E. coli* JMpβ5 as described by Date *et al*. [28]. The human pol γ catalytic gene was cloned into pFastBac. Histidine-tagged enzyme was expressed using the BAC-TO-BAC HT Baculovirus Expression System according to the supplier's manual (LIFE TECHNOLOGIES, MD, USA) and purified using ProBoundresin (Invitrogen Japan, Tokyo, Japan) [29]. Human pols δ and ε were purified by the nuclear fractionation of human peripheral blood cancer cells (Molt-4) using the second subunit of pols δ and ε-conjugated affinity column chromatography, respectively [30]. Recombinant human pols η and ι tagged with His_6_ at their C-terminal were expressed in SF9 insect cells using the baculovirus expression system, and were purified as described previously [31, 32]. A truncated form of pol κ (i.e., hDINB1DC) with 6 x His-tags attached at the C-terminal was overproduced using the BAC-to-BAC Baculovirus Expression System kit (GIBCO BRL, MD, USA) and purified as described previously [33]. Recombinant human His-pol λ was overexpressed and purified according to a method described previously [34]. Fish pol δ was purified from the testis of cherry salmon (*Oncorhynchus masou*) [35]. Pol I (α-like) from a higher plant, cauliflower inflorescence, was purified according to the methods outlined by Sakaguchi *et al*. [36]. The Klenow fragment of pol I from *E. coli* was purchased from Worthington Biochemical Corp. (Freehold, NJ, USA). *Taq* pol, T4 pol, T7 RNA polymerase and T4 polynucleotide kinase were purchased from Takara (Kyoto, Japan). Bovine pancreas deoxyribonuclease I (DNase I) was obtained from Stratagene Cloning Systems (La Jolla, CA, USA). 

### DNA polymerase assays

The reaction mixtures for pol α, pol β, fish pol δ, plant pol I (α-like) and prokaryotic pols were described previously [37, 38], and those for pol γ, and pols δ and ε were as described by Umeda *et al*. [29] and Ogawa *et al*. [39], respectively. The reaction mixtures for pols η, ι and κ were the same as for pol α, and the reaction mixture for pol λ was the same as that for pol β. 

For pols, poly(dA)/oligo(dT)_12-18_ (A/T = 2/1) and dTTP were used as the DNA template-primer and nucleotide (i.e., dNTP) substrate, respectively. KA-A analogues (i.e., compounds **1**–**12**) were dissolved in distilled dimethyl sulfoxide (DMSO) at various concentrations and sonicated for 30 sec. Aliquots of 4 μL of sonicated samples were mixed with 16 μL of each enzyme (final amount 0.05 units) in 50 mM Tris-HCl (pH 7.5) containing 1 mM dithiothreitol, 50% glycerol and 0.1 mM EDTA, and kept at 0 °C for 10 min. These inhibitor-enzyme mixtures (8 μl) were added to 16 μL of each of the enzyme standard reaction mixtures, and incubation was carried out at 37 °C for 60 min, except for *Taq* pol, which was incubated at 74 °C for 60 min. Activity without the inhibitor was considered 100%, and the remaining activity at each concentration of the inhibitor was determined relative to this value. One unit of pol activity was defined as the amount of enzyme that catalyzed the incorporation of 1 nmol of dNTP (i.e., dTTP) into synthetic DNA template-primers in 60 min at 37 °C under the normal reaction conditions for each enzyme [37, 38]. 

### Investigation of growth rate on cultured human cancer cells

To investigate the effects of KA-A derivatives in cultured cells, we used a human cancer cell line, HL-60, human promyelocytic leukemia cells (IFO 050022), supplied by the Health Science Research Resources Bank (Osaka, Japan). The cells were routinely cultured in RPMI 1640 medium supplemented with 10% fetal bovine serum, 100 μg/mL streptomycin, 100 unit/mL penicillin, and 1.6 mg/mL NaHCO_3_. The cells were cultured at 37 °C in standard medium in a humidified atmosphere of 5% CO_2_-95% air. The rate of the cancer cell growth of the compound was investigated as follows: high concentrations (10 mM) of the compounds were dissolved in DMSO and stocked. Approximately 1 x 10^4^ cells per well were inoculated in 96-well micro plates, and then the compound stock solution was diluted to various concentrations, and applied to each well. After incubation for 24 hr, the survival rate was determined by MTT (3-(4,5-dimethylthiazol-2-yl)-2,5-diphenyl tetrazolium bromide) assay [40]. 

### Other enzyme assays

The primase activity of calf pol α, the activities of T7 RNA polymerase, T4 polynucleotide kinase and bovine DNase I were measured in standard assays according to the manufacturer's specifications as described by Tamiya-Koizumi *et al*. [41], Nakayama *et al*. [42], Soltis *et al*. [43], and Lu and Sakaguchi [44], respectively. 

### KA-A derivatives docking modeling

The molecular docking of KA-A derivatives and the 8-kDa domain of pol β (Protein Data Bank (PDB) code: 1BNO and 1BPD) was performed using a fixed docking procedure in the Affinity program of Insight II modeling software (Accerlys Inc., San Diego, CA, USA, 1999). The calculations used a CVFF force-field in the Discovery program and a Monte Carlo strategy in the Affinity program [45]. Each energy-minimized final docking position of KA-A derivatives was evaluated using the interactive score function in the Ludi module. The Ludi score includes the contribution of the loss of translational and rotational entropy of the fragment, the number and quality of hydrogen bonds, and contributions from ionic and lipophilic interactions to the binding energy. 
